# Towards scaling up the sonochemical synthesis of Pt-nanocatalysts

**DOI:** 10.1016/j.ultsonch.2024.106794

**Published:** 2024-02-05

**Authors:** Henrik E. Hansen, Thea B. Berge, Frode Seland, Svein Sunde, Odne S. Burheim, Bruno G. Pollet

**Affiliations:** a Electrochemistry Group, Department of Materials Science and Engineering, Faculty of Natural Sciences. Norwegian University of Science and Technology (NTNU), NO-7491 Trondheim, Norway; b Department of Energy and Process Engineering, Faculty of Engineering, Norwegian University of Science and Technology (NTNU), NO-7491 Trondheim, Norway; c Green Hydrogen Lab, Institute for Hydrogen Research (IHR), Université Du Québec à Trois-Rivières (UQTR), 3351 Boulevard des Forges, Trois-Rivières, Québec G9A 5H7, Canada

**Keywords:** Scale-up, Electrocatalysts, Sonochemistry, Fuel cells, ORR, Pt, Nanoparticle synthesis

## Abstract

Large scale production of electrocatalysts for electrochemical energy conversion devices such as proton exchange membrane fuel cells must be developed to reduce their cost. The current chemical reduction methods used for this synthesis suffer from problems with achieving similar particle properties such as particle size and catalytic activity when scaling up the volume or the precursor concentration. The continuous production of reducing agents through the sonochemical synthesis method could help maintain the reducing conditions (and also the particle properties) upon increasing the reactor volume. In this work we demonstrate that the reducing conditions of Pt-nanoparticles are indeed maintained when the reactor volume is increased from 200 mL to 800 mL. Similar particle sizes, 2.1(0.3) nm at 200 mL and 2.3(0.4) nm at 800 mL, and catalytic activities towards the oxygen reduction reaction (ORR) are maintained as well. The reducing conditions were assessed through TiOSO_4_ dosimetry, sonochemiluminesence imaging, acoustic power measurements, and Pt(II) reduction rate measurements. Cyclic voltammetry, CO-stripping, hydrogen evolution measurements, ORR measurements, and electron microscopy were used to evaluate the catalytic activity and particle size. The similar particle properties displayed from the two reactor volumes suggest that the sonochemical synthesis of Pt-nanoparticles is suitable for large scale production.

## 1 Introduction

Large scale production of proton exchange membrane fuel cells (PEMFC) is important for realizing a hydrogen economy [1], [2]. However, challenges related to the durability and production cost of the catalysts and catalyst layer need to be resolved [1]. A PEMFC generates useful electrical energy from hydrogen and oxygen through electrochemical reactions. The largest contribution to the overall voltage loss in a PEMFC is the slow kinetics of the oxygen reduction reaction (ORR) occurring at the cathode [1].

(1) $$O_{2}+4H^{+}+4e^{-}⟶2H_{2}O$$

A higher catalyst loading is therefore needed at the cathode in order to achieve the same activity as on the anode while maintaining a reasonably low overpotential. This leads to a higher cost of the fuel cell. The loading can be reduced if the intrinsic activity of the ORR catalysts is improved. Much effort has been devoted to developing high performance ORR catalysts through alloying, core–shell structures, and other novel catalyst designs [3], [4], [5], [6]. However, in order for large scale production to be realized, the catalyst must retain its performance when the synthesis method is scaled up.

Current nanocatalyst synthesis methods based on fast chemical reduction struggle with reproducibility when key parameters such as the volume or precursor concentrations are increased [7], [8]. In addition, chemical reduction methods rely on the addition of reducing agents to form the nanocatalyst. This makes chemical reduction more difficult to automate compared to a continuous synthesis method.

The sonochemical synthesis method offers an alternative way of producing nanocatalysts without the addition of reducing agents. Instead, the reducing agents are generated in situ through sonolysis of water.

(2) $$(H_{2}O))\cdot \mathrm{OH}+\cdot H$$

Increasing the reactor height (and volume) is an important step towards large scale nanoparticle production through the sonochemical method. If the nanoparticle properties can be maintained while the solution volume is increased, it could offer much more flexibility than traditional chemical reduction methods. As a result, small scale studies would also be applicable on larger scales, which can save time and resources in the development of new catalyst materials.

If the nanoparticle properties are to be maintained when scaling up the synthesis, the reduction conditions provided by the number of primary radicals also need to be maintained. Previous investigations into the effect of solution volume on radical formation have shown that similar amounts of radicals are generated for different reactor volumes [9], [10]. Asakura et al. [9] showed that the formation rate of $I_{3}^{-}$ at 514 kHz was the same for volumes between 25 and 200 mL provided the acoustic power was kept below the sonochemical quenching limit for the given volume. At 200 mL they observed that sonochemical quenching started at 90 W with lower volumes displaying lower quenching limits. Son et al. [10] performed similar experiments for much larger reactor sizes with volumes ranging from 0.47 L to 2.82 L using ultrasonic frequencies of 36 kHz and 108 kHz with acoustic powers of 33.5(1.4)W and 38.3(1.6)W for the respective frequencies. Upon increasing the volume they found a slight increase in the formation rate of $I_{3}^{-}$ at 36 kHz. For 108 kHz, the formation rate was similar for all volumes.

As the number of primary radicals presented in the aforementioned works appears to be somewhat constant with increasing reactor volume, the sonochemical synthesis conditions of nanoparticles should also remain unchanged upon increasing the reactor volume. The resulting nanoparticles should therefore also exhibit similar particle properties, which would make the sonochemical synthesis method well suited for large scale nanoparticle synthesis.

In this work we have assessed the scale-up potential of the sonochemical method with regards to reproducibility through the sonochemical synthesis of Pt-nanoparticles at low (200 mL) and high (800 mL) reactor volumes. This was evaluated by comparing the Pt-nanoparticle size and electrochemical characteristics towards the oxygen reduction reaction, CO-oxidation, and hydrogen evolution reaction resulting from the two reactor volumes. The reduction rates of the Pt(IV)/Pt(II) precursor as measured from UV–visible spectroscopy, dosimetric detection of primary radicals, and calorimetric measurements of the acoustic power were used to evaluate the sonochemical reduction conditions for the two reactor volumes.

## 2 Experimental

### 2.1 Chemicals

Ethanol (EtOH) (VWR, 96% GPR RECTAPUR), platinum tetrachloride (PtCl_4_) (Sigma Aldrich, 96%), titanium(IV) oxysulfate solution (TiOSO_4_) (Sigma Aldrich, 1.9-2.1%), and potassium iodide (KI) (Sigma Aldrich, ⩾99% ACS reagent), Nafion 117 (Sigma Aldrich, 5% in a mixture of lower aliphatic alcohols and water), sulfuric acid (H_2_SO_4_) (Sigma Aldrich, 96%), isopropyl alcohol (IPA) (VWR, technical), carbon monoxide (CO) (4.7), oxygen (O_2_) (5.0), argon (Ar) (5.0), luminol (Sigma Aldrich, ⩾97%), sodium hydroxide (NaOH) (Sigma Aldrich, reagent grade ⩾98%), VULCAN XC-72 carbon black (Cabot Corp.) were used as received from the supplier. Milli-Q water (18.2 MΩ·cm) was used for all experiments.

### 2.2 Sonochemical Setup

**Fig. 1:**
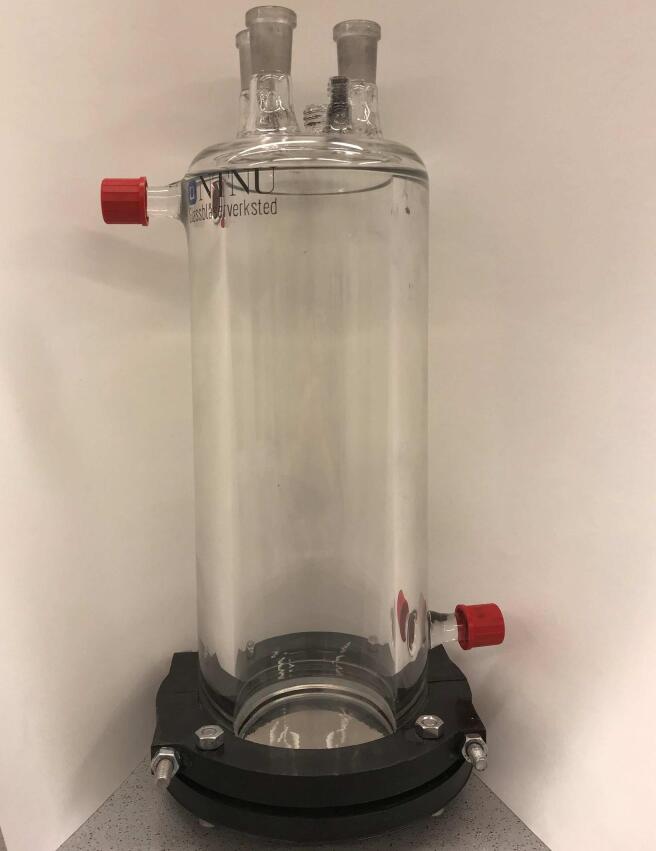
The sonochemical reactor used for all experiments. The ultrasonic transducer makes up the bottom of the reactor. Ports at the top allows for introduction of gas and sample extraction. Water can be circulated through the external cooling jacket, where the inlet and outlet are located at the bottom right and upper left, respectively.

The sonochemical reactor used for all experiments is shown in Fig. 1. A Honda Electronics 346 kHz lead zirconate titanate (PZT) ultrasonic transducer with a stainless steel (SUS304) plate (ø 70 mm) was located at the bottom of the reactor in direct contact with the precursor solution. Contrary to low frequency (20 kHz) ultrasound systems, higher ultrasonic frequencies, such as 346 kHz, have previously been shown to not cause plate erosion [11]. The reactor was fitted with an external circulating water cooling jacket to maintain a constant temperature of approximately 3°C inside the reactor. The ultrasonic signals were generated with an AG 1012 RF signal generator in combination with an impedance matching unit (T1k-7A) from T&C Power Conversion. A constant electric power of 50 W was applied to the plate transducer for all experiments. Calorimetric experiments revealed that the acoustic power transferred to the sonochemical reactor was 42(1)W. All solutions were purged with Ar (5.0) prior to and throughout sonication in order to prevent atmospheric gasses from altering the collapse conditions of the cavitation bubbles.

### 2.3 Ultrasound Characterization

Calorimetric and dosimetric measurements of the sonochemical reactor were performed for different solution volumes between 200 mL and 900 mL to assess the impact of solution volume on the acoustic power and radical generation, respectively. For the calorimetric measurements the temperature increase of room-temperature water was measured during sonication over a period of two minutes. The temperature was measured with a Fluke 289/287 type-K thermocouple. The slope of the resulting linear temperature increase (d*T*/d*t*) was then used to calculate the acoustic power ($P_{a}$)

(3) $$P_{a}=\rho {\mathrm{VC}}_{p}\left(\frac{\mathrm{dT}}{\mathrm{dt}}\right)$$

where ρ is the water density (0.998 kg dm^−3^ at 20°C), *V* is the water volume, and $C_{p}$ is the specific heat capacity of water at constant pressure (4.186 J g^−1^ K^−1^). Three replicate experiments were conducted for each volume.

For the dosimetric measurements, hydrogen peroxide resulting from ·OH radical recombination in water was measured by UV–visible spectroscopy (UV–vis). Hydrogen peroxide detection by UV–vis is achieved through addition of excess TiOSO_4_ which creates a characteristic yellow colour with an absorbance peak at 411 nm (∊ = 787 mol^−1^dm^3^ cm^−1^) [12]. Aliquots of the solution were therefore mixed with TiOSO_4_ at different sonication times to assess the development of the ·OH radical generation over a period of 20 min. The slope of the resulting linear curves were then used to quantify the ·OH radical generation rate for solution volumes between 200 mL and 900 mL. Three replicate experiments were performed at 200 mL, 800 mL, and 900 mL.

### 2.4 Sonochemical Synthesis

In order to evaluate if the sonochemical method is suitable for large scale production, 1mmol dm^−3^ PtCl_4_ solutions were prepared in volumes of 200 mL and 800 mL. The resulting samples from these experiments will be referred to as the 200 mL sample, and the 800 mL sample. 0.5 mol dm^−3^ ethanol was used as the radical scavenger for both experiments following preliminary experiments on the optimal ethanol concentration for sonochemical synthesis of Pt-nanoparticles (Fig. S1). This ethanol concentration is also in line with results from our previous work [13]. The precursor solutions were then sonicated until a pitch-black solution was obtained at which point XC-72 vulcan carbon was added to the solution. The addition of carbon towards the end of the synthesis was performed as the presence of carbon during sonication was shown to severely impact the rate of reduction (Fig. S2). The amount of carbon added to each solution was estimated to yield a Pt-loading of 20wt% assuming complete Pt-reduction. To allow the catalyst to be dispersed well onto the carbon support sonication was continued for 15 min after adding the carbon.

The resulting colloidal dispersion of Pt/C was then filtered through a Supor 800 0.8μm 90 mm membrane filter from Pall Corp. and cleaned three times with water and ethanol. The particles were then dried in air before being crushed into a fine powder with a mortar and pestle.

### 2.5 Colorimetric Detection of Pt(IV) and Pt(II)

The concentration of Pt(IV) and Pt(II) were assessed colorimetrically at select sonication times by mixing aliquots of the sonicated Pt-solution with an excess of KI. The resulting ${\mathrm{PtI}}_{6}^{2-}$ and ${\mathrm{PtI}}_{4}^{2-}$ complexes corresponding to Pt(IV) and Pt(II), respectively, display intense peaks in the UV–vis spectrum; 495 nm (∊ = 9400 mol^−1^dm^3^ cm^−1^) for Pt(IV) and 388 nm (∊ = 4600 mol^−1^dm^3^ cm^−1^) for Pt(II) [14], [15]. Contributions from any Pt-nanoparticles were removed by filtering the samples through a 0.2 μm PTFE acrodisc syringe filter prior UV–vis measurements [15]. Detection of Pt(II) does suffer from interference from the Pt(IV) peak and the contribution from the Pt(IV) peak must therefore be removed to accurately assess the development of Pt(II) in solution. A detailed explanation on how this procedure is performed can be found in our previous work [12].

### 2.6 Electrochemical Characterization

The catalytic activity of the catalysts towards the hydrogen evolution reaction (HER) and the oxygen reduction reaction (ORR) was assessed through linear sweep voltammetry (LSV). The electrochemical active surface area (ECSA) was evaluated through hydrogen underpotential deposition (UPD) and CO-stripping voltammetry.

Electrochemical characterization was performed in a three electrode setup containing Ar-saturated 0.5 mol dm^−3^ H_2_SO_4_. A glassy carbon rotating disk electrode (A  = 0.196 cm^2^) coated with the catalyst ink was used as the working electrode. A reversible hydrogen electrode (RHE) was used as the reference electrode, and all voltages are reported against the RHE. A graphite rod was used as the counter electrode. The catalyst ink was prepared by mixing 10 mg catalyst with 475 μL water and 475 μL isopropyl alcohol before adding 50 μL Nafion. 10 μL of the ink was then drop cast on the glassy carbon electrode to achieve a target catalyst loading of 0.1 mg cm^−2^. However, the actual Pt-loading was lower than this due to incomplete reduction of the Pt-precursors during the synthesis (Fig. 4) and and was found to be 0.1 mg cm^−2^ and 0.05 mg cm^−2^ for the 200 mL and 800 mL samples, respectively.

Prior to the electrochemical measurements the catalyst was preconditioned through cyclic voltammetry between 0.02–1.5 V at 50 mV s^−1^ for 20 cycles at which point the characteristic Pt voltammogram had remained stable for multiple cycles. The final cycle was used to determine the ECSA through integration of the hydrogen adsorption UPD peaks (0.06–0.35 V) in the voltammograms using a hydrogen charge density of 220 μC cm^−2^ [16]. Linear sweep voltammetry in the HER region was performed at 1 mV s^−1^ under rotation at 1600 rpm, and the resulting current was normalized for the ECSA of the respective catalysts. For the ORR experiments, the electrolyte was bubbled with O_2_ for 20 min before linear sweep voltammetry was conducted from 1.2 V to 0.02 V with a scan rate of 1 mV s^−1^ and a rotation rate of 1600 rpm.

For the CO-stripping experiments a new ink was deposited and preconditioned under Ar-saturated 0.5 mol dm^−3^ H_2_SO_4_ through cyclic voltammetry. CVs were recorded between 0.02–1.5 V at 50 mV s^−1^ for 150 cycles following the methodology described by Svendby et al. [17]. The electrolyte was then saturated with CO under a constant applied potential of 0.05 V to allow for CO to adsorb to the catalyst surface. Unadsorbed CO was then removed from the electrolyte through Ar-purging. The CO-stripping was performed by cycling the potential between 0.05–1.2 V at 10 mV s^−1^. The ECSA obtained from the CO-stripping peak was found by integrating the area between the first CO-stripping cycle and the subsequent cycle using a charge density of 440 μC cm^−2^.

### 2.7 Electron Microscopy

Nanoparticle imaging was conducted with a Hitachi High-Tech SU9000 electron microscope in an ISO 7 cleanroom. Samples were prepared by drop casting 5 μL nanoparticles dispersed in ethanol and water onto a Formvar, carbon-coated Cu TEM-grid. Images were acquired in secondary electron mode and brightfield mode with an acceleration voltage of 30 kV and an emission current of 4.7 μA. Particle size measurements of the resulting images were performed using ImageJ. Over 200 individual particles were measured manually for each sample.

### 2.8 Sonochemiluminesence Experiments

The distribution of radicals throughout the sonoreactor at 200 mL and 800 mL was assessed through sonochemiluminesence imaging. This method is described by Son et al. [10]. 0.1 g L^−1^ luminol was mixed with 1 g L^−1^ NaOH in Milli-Q water. A Canon EOS 500D with a 50 mm lens was used to acquire long exposure images of the luminol reacting with the ·OH radicals during sonication. The shutter speed was 30 s, and efforts were made to ensure completely dark surroundings during imaging.

## 3 Results

**Fig. 2:**
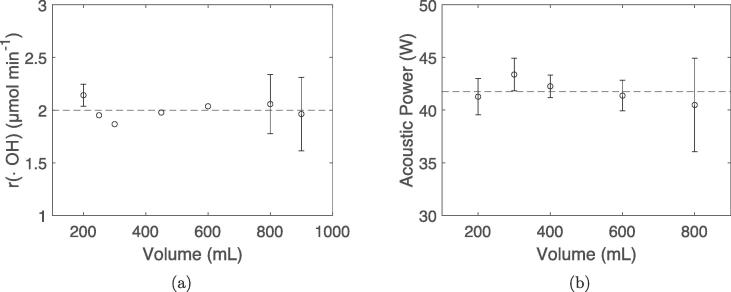
Rate of ·OH radical formation (a) and acoustic power (b) as a function of reactor volume. Mean values for all volumes are indicated by the dotted lines. UV–vis spectra (Fig. S3) and temperature profiles (Fig. S4) are provided in the supporting information.

The ·OH radical formation rate is fairly constant for all reactor volumes with a mean value of 2.0(0.25) μmol min^−1^ (Fig. 2a). However, more variable results are observed at higher volumes. No significant differences in acoustic power can be observed for the different reactor volumes (Fig. 2b), although the 800 mL reactor volume exhibits more variable acoustic power than the other volumes. The mean acoustic power for all reactor volumes was found to be 42(1) W.

**Fig. 3:**
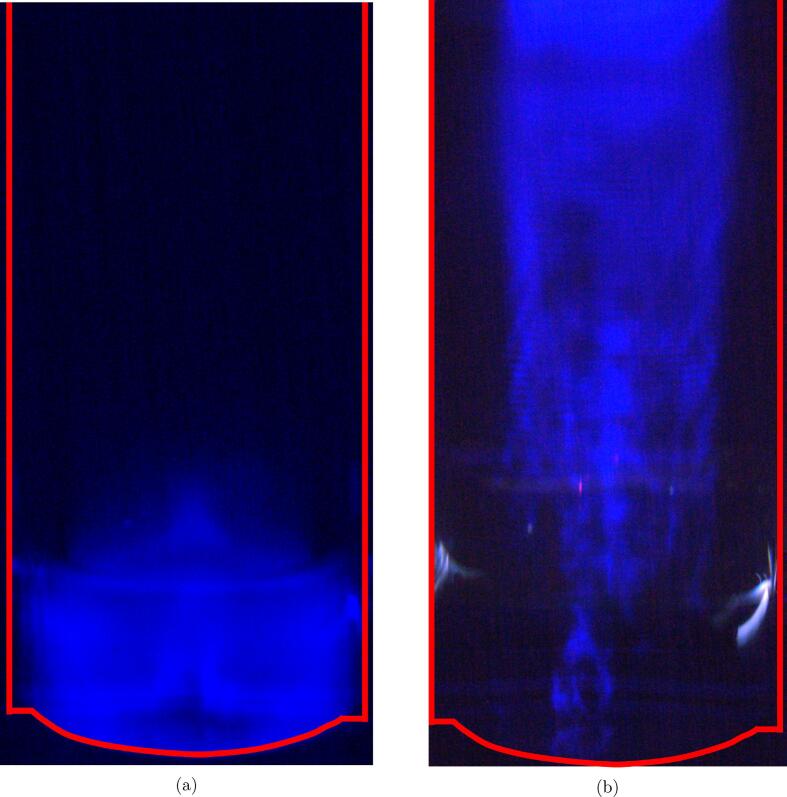
Sonochemiluminesence images of a 200 mL (a) and 800 mL (b) sonoreactor. The outer reactor walls and the transducer at the bottom of the reactor are outlined in red. The blue colour represents areas of sonochemical activity.

The cavitation field obtained from different reactor volumes is visualized by sonochemiluminesence images in Fig. 3. The blue areas indicate regions where ·OH radicals react with luminol, and therefore provide an indication of where the ·OH radicals are generated. For a reactor volume of 200 mL, a fairly constant colour is observed throughout the solution volume. For a reactor volume of 800 mL, a noticeable difference is observed between the upper and lower parts of the reactor. Close to the transducer, very little activity can be seen. Moving closer to the solution surface, distinct blue lines can be seen before a constant blue colour appears near the surface. However, water samples extracted from different parts of the reactor showed that there is no significant difference in radical concentration if samples are extracted close to the transducer, at the center of the reactor, or near the surface of the solution (Fig. S5). This suggests that the radicals are well mixed for both reactor volumes.

**Fig. 4:**
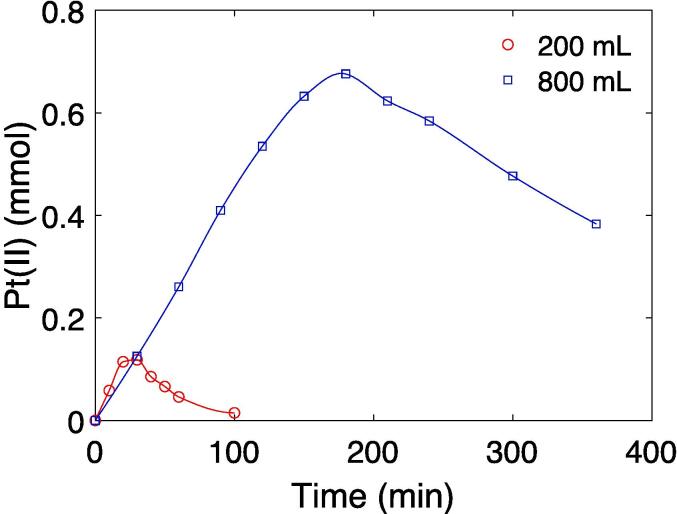
Formation of Pt(II) as a function of sonication times at different reaction volumes. The initial PtCl_4_ concentration was 1 mmol dm^−3^. UV–vis spectra of the Pt(IV)/Pt(II) samples from the two volumes are given in the supporting information (Fig. S6).

The amount of Pt(II) obtained as a function of sonication time at different reaction volumes and equal precursor concentrations is shown in Fig. 4. The maximum in the amount of Pt(II) displayed by these curves arises due to the initial formation of Pt(II) through the reduction of Pt(IV) followed by a reduction of Pt(II) to Pt-nanoparticles. Pt-nanoparticle formation has been shown to begin around the maximum in the Pt(II) amount [14]. The rates of Pt(II) and Pt-nanoparticle formation can therefore be estimated from the slopes before and after the maximum Pt(II) amount in Fig. 4, respectively. The rate of Pt(II) formation was found to be 4.8 μmol min^−1^ at 200 mL and 3.8 μmol min^−1^) at 800 mL. The following reduction of Pt(II) to Pt-nanoparticles was slower, but it gave comparable reduction rates at 200 mL (1.65 μmol min^−1^) and 800 mL (1.34 μmol min^−1^). Comparing the measured Pt(II) amount to the maximum obtainable amount of Pt(II), an estimated 94% yield of Pt-nanoparticles was obtained for the 200 mL sample. For the 800 mL sample, an estimated 50% yield of Pt-nanoparticles was obtained. The Pt loading on the carbon support can therefore be estimated to be about 20% and 10% for the 200 mL and 800 mL sample, respectively. A comparison between the different Pt-nanoparticle characteristics at 200 mL and 800 mL is shown in Table 1.

**Table 1:** Comparison table for the Pt-nanoparticles synthesized with a reactor volume of 200 mL and 800 mL. Pt(II)-rate and Pt(0) rate represent the rate at which Pt(II) and Pt-nanoparticles are formed, respectively. Pt-loading represents the loading of Pt-nanoparticles on the carbon support as estimated from the Pt-nanoparticle yield. ECSA (H) and ECSA (CO) represents the electrochemical active surface area from hydrogen underpotential deposition and CO-stripping, respectively. The CO-peak is the main CO-stripping peak, and the particle size represents the nanoparticle size as obtained from electron microscopy.

	200 mL	800 mL
Pt(II) rate	4.8 μmol min^−1^	3.8 μmol min^−1^
Pt(0) rate	1.65 μmol min^−1^	1.34 μmol min^−1^
Pt-loading	20%	10%
ECSA (H)	94 ± 2 m^2^g^−1^	97 ± 1 m^2^g^−1^
ECSA (CO)	102 m^2^g^−1^	105 m^2^g^−1^
CO-peak	0.802 V	0.800 V
Particle size	2.3 ± 0.4 nm	2.1 ± 0.3 nm

**Fig. 5:**
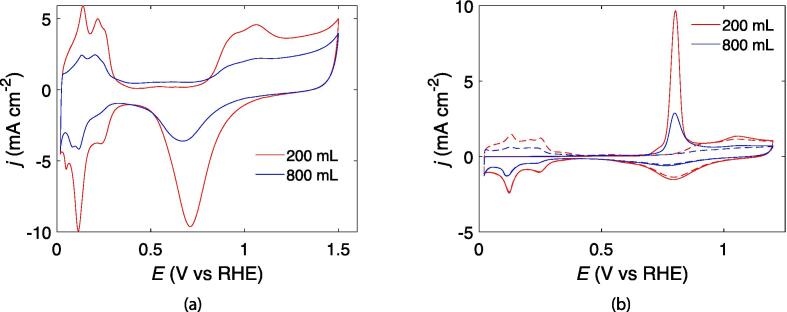
Cyclic voltammograms of Pt/C (20%) in 0.5 mol dm^−3^ H_2_SO_4_ under Ar atmosphere with a scan rate of 50 mV s^−1^ (a) and during CO-stripping with a scan rate of 10 mV s^−1^ (b). The subsequent cycle after the CO-stripping is indicated by a dotted line.

The voltammograms of the Pt catalysts in sulfuric acid (Fig. 5a) resembles all the expected features; the hydrogen UPD region is seen at low potentials and Pt oxide formation and reduction at higher potentials. The CO-stripping voltammograms (Fig. 5b) are also as expected for a monolayer of adsorbed CO at a Pt electrode, with a stripping peak potential of about 0.8 V [17]. From the hydrogen UPD peaks in Fig. 5, Fig. 5b the ECSA was found to be 97(1) m^2^g^−1^ and 94(2) m^2^g^−1^ for the 800 mL and 200 mL samples, respectively. The CO-stripping peaks in Fig. 5b revealed an ECSA of 105 m^2^g^−1^ and 102 m^2^g^−1^ for the 800 mL and 200 mL samples, respectively. Both ECSA estimates were calculated assuming a Pt-loading of 20% and 10% for the 200 mL and 800 mL sample, respectively. The main CO-peak in Fig. 5b was observed at peak potentials of 0.800 V and 0.802 V for the 800 mL and 200 mL samples, respectively.

**Fig. 6:**
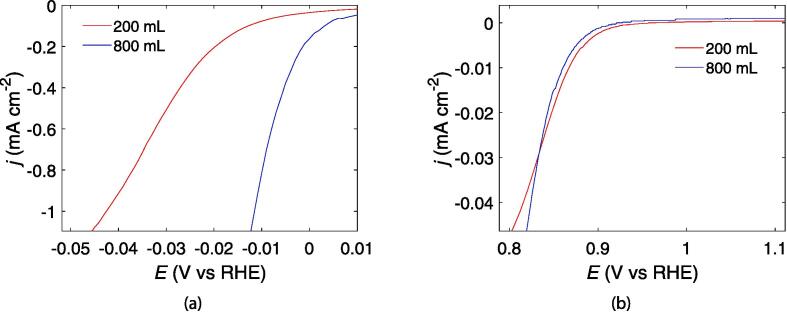
Polarization curves of Pt/C (20%) in 0.5 mol dm^−3^ H_2_SO_4_ under Ar atmosphere for the hydrogen evolution reaction (a), and under O_2_ atmosphere for the oxygen reduction reaction (b). The currents are normalized for the electrochemical active surface area from the hydrogen underpotential deposition peaks of the respective catalysts.

Polarization curves for the HER and ORR of the Pt/C synthesized with different reactor volumes are shown in Fig. 6, Fig. 6, respectively. The current is normalized for the ECSA of the respective samples. Both samples display very high HER activities as is characteristic for Pt-nanoparticles. However, the 800 mL sample shows a much higher HER activity than the 200 mL sample. For the ORR activity, the two samples behave much more similarly.

**Fig. 7:**
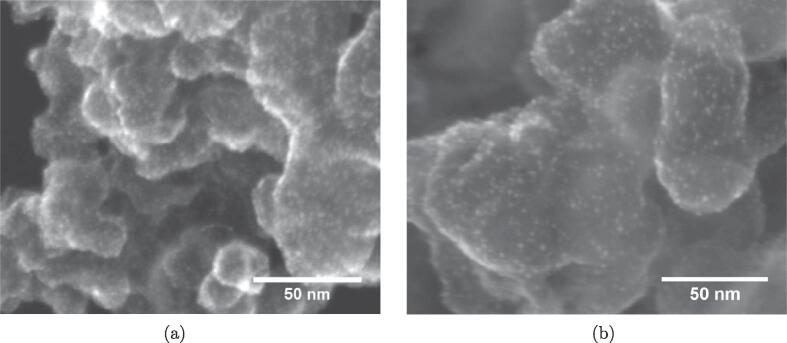
Secondary electron microscopy images of Pt/C sonochemically synthesized with a reactor volume of 200 mL (a) and 800 mL (b).

The particle sizes from electron microscopy images (Fig. 7) were found to be 2.1(0.3) nm and 2.3(0.4) nm for the 800 mL sample and the 200 mL sample, respectively. The Pt-nanoparticles appear to be well dispersed on the carbon support for both samples. Qualitative assessment of the particle dispersion revealed a higher Pt-loading on the 200 mL sample compared to the 800 mL sample.

## 4 Discussion

The sonochemical reduction conditions of Pt-nanoparticles appear to be quite similar when scaling up the sonochemical reactor from 200 mL to 800 mL. This is reflected in the fairly constant primary radical generation (Fig. 2a) and acoustic power (Fig. 2b), as well as similar reduction rates of Pt(II) to Pt-nanoparticles (Fig. 4) for the different reactor volumes. Similar particle sizes (Fig. 7), ORR activities (Fig. 6b), and CO-stripping peak positions (Fig. 5b) also reveal that the resulting particle properties are very similar when scaling up the volume. Achieving such reproducible particle properties when scaling up the volume can be a challenge in traditional chemical reduction methods [7], [8].

With the radical generation being the driving force for nanoparticle formation, the similar radical generation rates, 2.0(0.25) μmol min^−1^, and acoustic powers, 42(1) W, are good indicators that the reducing conditions for Pt-nanoparticles are similar as well. The reduction rates of Pt(II) to Pt-nanoparticles also display similar values for the 200 mL (1.65 μmol min^−1^) and 800 mL (1.34 μmol min^−1^) reactor. This is expected as the Pt(II) reduction is driven mostly by secondary radicals, which is proportional to the primary radical generation [14], [18]. Based on previous radiolytic investigations by Asmus et al. 84.3% of hydrogen abstraction by primary radicals occurs at the alpha carbon in ethanol, which is the only secondary radical capable of electron donation [19]. The observed primary radical generation rates and the Pt(II) reduction rates are therefore in good agreement with the expected hydrogen abstraction probabilities for ethanol. The similar sonochemical synthesis conditions can therefore be traced back to the radical generation as expected.

These similar synthesis conditions are also reflected in the resulting particle properties obtained for the two sonoreactor volumes. Both sonoreactor volumes appear to yield fairly monodisperse Pt-nanoparticles as seen from the electron microscopy images in Fig. 7. The similar particle sizes are also further supported by the similar ORR activity displayed by the 200 mL and 800 mL samples in the kinetic region of the voltammogram (Fig. 6b). The ORR activity has been shown to be dependent on the size of Pt-nanoparticles with optimum activities being achieved for particle sizes of around 3 nm [5], [4], [6], [3]. Furthermore, both the 200 mL and 800 mL sample display identical positions for their CO-stripping peaks (Fig. 5b). For Pt-nanoparticles, the potential of the CO-stripping peak has been shown to be highly dependent on the particle size with smaller particles displaying more positive peak potentials [20], [21].

Despite the similar reduction rates, acoustic powers, radical formation rates and particle properties resulting from the two reactor volumes, there are some small differences in the cavitation fields close to the transducers as shown from sonochemiluminesence images (Fig. 3). However, it appears that the acoustic streaming ensures sufficient mixing to allow for a homogeneous distribution of radicals throughout the 800 mL reactor as indicated in Fig. S5. Still, the uneven cavitation field at higher volumes may give rise to the slightly higher standard deviations observed for the radical formation and acoustic power at 800 mL. Evidently, this does not affect the final Pt-nanoparticle properties in this work.

Further examination of the radical yield and acoustic power suggests that the number of cavitation bubbles produced in a sonoreactor is independent of the reactor volume. Both the acoustic power and the number of radicals being formed are dependent on the collapse conditions of single cavitation bubbles and how many cavitation bubbles collapse. Assuming a simple adiabatic collapse of the cavitation bubble, the radical generation and the measured acoustic power is proportional to the collapse temperature (*T*), which is given as

(4) $$T=T_{\infty }{\left(\frac{R}{R_{0}}\right)}^{\left(\gamma -1\right)}$$

where $T_{\infty }$ is the bulk temperature, *R* is the collapse radius of the cavitation bubble, $R_{0}$ is the ambient radius of the bubble, and γ is the polytropic ratio of the gas inside the bubble [22]. None of these parameters are significantly dependent on the reactor volume, which means that the same amount of radicals is expected to be produced per cavitation bubble for different reactor volumes.

The radical generation and the measured acoustic power is also proportional to the number of cavitation bubbles. A higher sonoreactor does display more antinodes than a lower sonoreactor, and therefore offers more sites for cavitation bubbles to produce primary radicals. However, if more cavitation bubbles are present for the 800 mL reactor compared to the 200 mL reactor, a higher acoustic power and radical yield would also be expected. From Fig. 2b and Fig. 2a both the acoustic power and number of radicals are constant when changing the solution volume (and height) which therefore suggests that the same number of active cavitation bubbles is present for both volumes.

The implication following volume independent generation of cavitation bubbles is therefore that the density of cavitation bubbles in the antinodes is higher at lower sonoreactor volumes. A higher density of cavitation bubbles means that the distance between individual cavitation bubbles (*d*) in the antinodes will be shorter as well. The secondary Bjerknes force ($F_{s}$) responsible for bubble coalescence is therefore expected to be higher at lower reactor volumes as shown through the following expression for the secondary Bjerknes force [23], [24], [9].

(5) $$F_{s}\propto {\left(\mathrm{fP}\right)}^{2}\left(\frac{V_{1}V_{2}}{d^{2}}\right)$$

*f* is the ultrasonic frequency, *P* is the acoustic power. $V_{1}$ and $V_{2}$ are the volumes of the two cavitation bubbles in question. A higher acoustic power threshold for sonochemical quenching should therefore be expected at higher reactor volumes [9]. Such a higher acoustic power threshold for higher reactor volumes was observed by Asakura et al. [9] for reactor volumes between 25 mL and 200 mL. The higher secondary Bjerknes forces observed for small reactor volumes by Asakura et al. further supports the idea that the density of cavitation bubbles is higher at lower sonoreactor volumes.

Large scale reactors could therefore potentially support much higher acoustic powers than small reactors due to the higher number of antinodes available for the cavitation bubbles. As a result, faster rates of nanoparticle formation could also be realized by transitioning to larger reactor sizes. Such a development would also require that the ultrasonic transducers are improved to withstand higher electrical power inputs.

Even though the synthesis conditions and the individual particle properties of the two samples appear to be similar, the catalyst loading differ. This is apparent from the remaining Pt(II) concentration at 800 mL compared to the complete reduction at 200 mL in Fig. 4. It can also be seen from the different currents in the cyclic voltammograms (Fig. 5) as well as visually from the electron microscope images (Fig. 7).

Extending the sonication time for the 800 mL sample would have been preferable to ensure equal loading, but legitimate concerns about operating the ultrasonic transducer close to the power limit for longer times prevented this. The different loading only appears to be significant for the HER activity with the higher loading for the 200 mL sample exhibiting a lower activity. However, lower HER activities are expected for higher Pt-loadings when the values are normalized for the ECSA [25]. The peak multiplicity observed in the CO-stripping voltammogram of the 200 mL sample is also an indication of a higher loading. The appearance of the small shoulder for the 200 mL sample suggests that the loading is higher compared to the 800 mL sample [26], [21]. For supported Pt-nanoparticles, the position of the main peak is not expected to be affected by the loading to any appreciable extent [26], [21].

## 5 Conclusions

Scaling up the sonochemical synthesis of Pt-nanoparticles from 200 mL to 800 mL retains the particle size and the catalytic activity towards the ORR. This is caused by similar reducing conditions brought about by a constant production of primary radicals when the solution volume is changed. As the particle sizes of the Pt-nanoparticles are also close to the optimal reported values for Pt ORR catalysts, the sonochemical synthesis of Pt-nanocatalysts may be scaled up without sacrificing catalyst performance, which is common in traditional chemical reduction methods. Additionally, increasing the reactor volume may also be a way to achieve higher primary radical formation rates as higher acoustic powers can be reached before sonochemical quenching begins.

## CRediT authorship contribution statement

**Henrik E. Hansen:** Conceptualization, Formal analysis, Investigation, Methodology, Visualization, Writing - original draft, Writing - review & editing. **Thea B. Berge:** Conceptualization, Data curation, Formal analysis, Investigation, Methodology, Validation, Writing - review & editing. **Frode Seland:** Supervision, Project administration, Validation, Writing - review & editing. **Svein Sunde:** Supervision, Validation, Writing - review & editing. **Odne S. Burheim:** Supervision, Validation, Resources, Funding acquisition, Writing - review & editing. **Bruno G. Pollet:** Supervision, Validation, Writing - review & editing.

## Declaration of Competing Interest

The authors declare that they have no known competing financial interests or personal relationships that could have appeared to influence the work reported in this paper.

## Data Availability

Data will be made available on request.
