# Supplementary material for: Towards scaling up the sonochemical synthesis of Pt-nanocatalysts
Source: Ultrason Sonochem. 2024 Feb 5;103:106794. doi: 10.1016/j.ultsonch.2024.106794 (PMC10878992; doi:10.1016/j.ultsonch.2024.106794)
Supplement: Supplementary data 1 [file mmc1.pdf]

Supporting Information

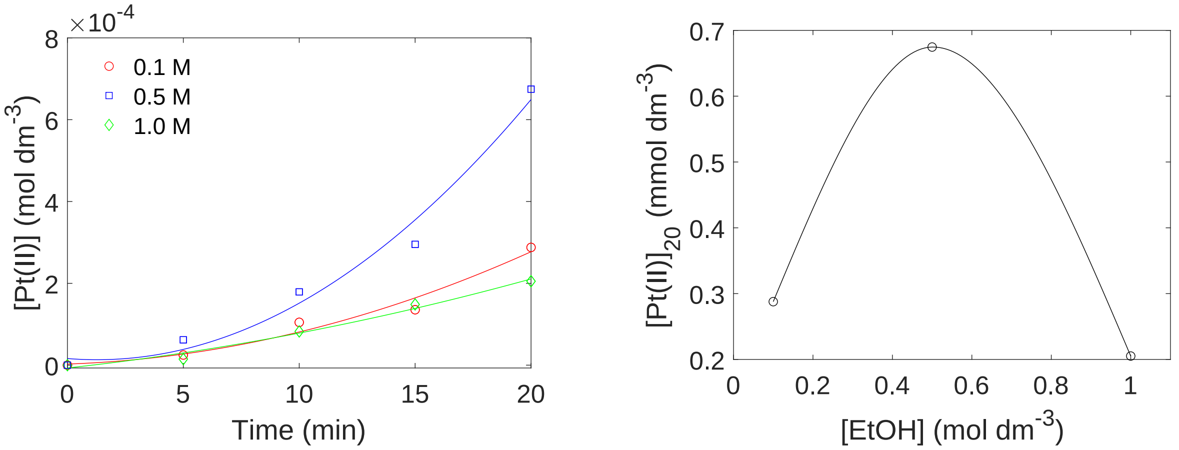

(a) (b)

Figure S1: Concentration of Pt(II) at different sonication times for ethanol scavenger concentrations of 0.1 mol dm^{-3} (○), 0.5 mol dm^{-3} (□), and 1.0 mol dm^{-3} (◇) (a). The Pt(II) concentration after 20 min of sonication is also plotted as a function of ethanol concentration (b).

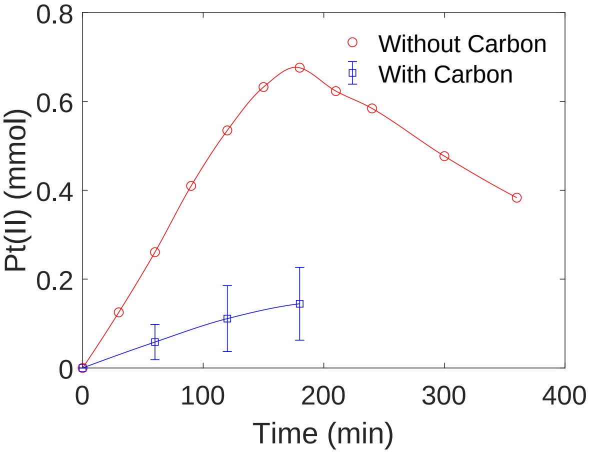

Figure S2: Concentration of Pt(II) as a function of sonication time where carbon was added towards the end of the synthesis (○) and at the beginning of the synthesis (□).

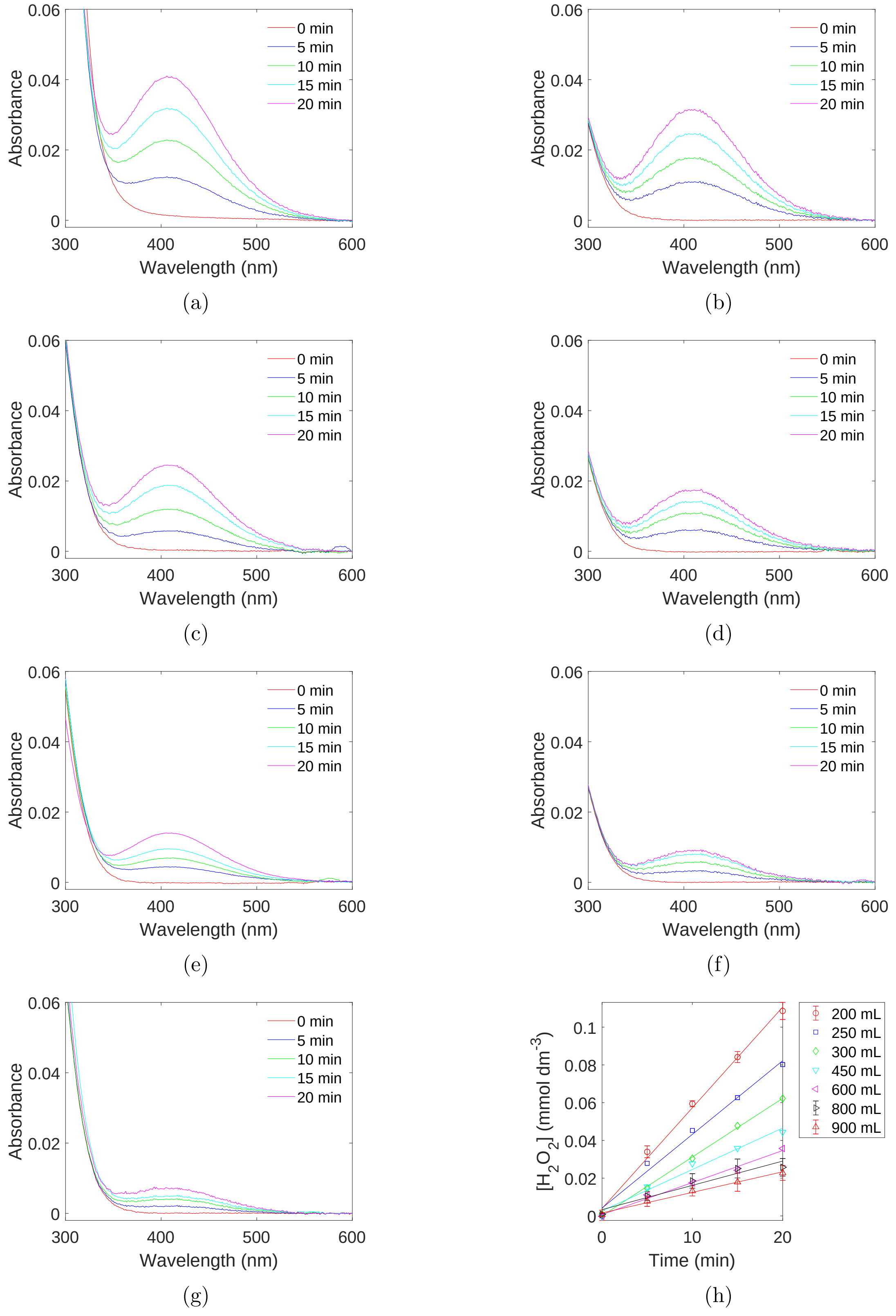

Figure S3: Absorbance spectra of the titanium(IV) peroxo complex used to estimate the H_2O_2 concentration at selected time intervals. Representative spectra are shown for reactor volumes of 200 mL (a), 250 mL (b), 300 mL (c), 450 mL (d), 600 mL (e), 800 mL (f), and 900 mL (g). The estimated H_2O_2 concentrations as a function of sonication time for all reactor volumes are also plotted (h).

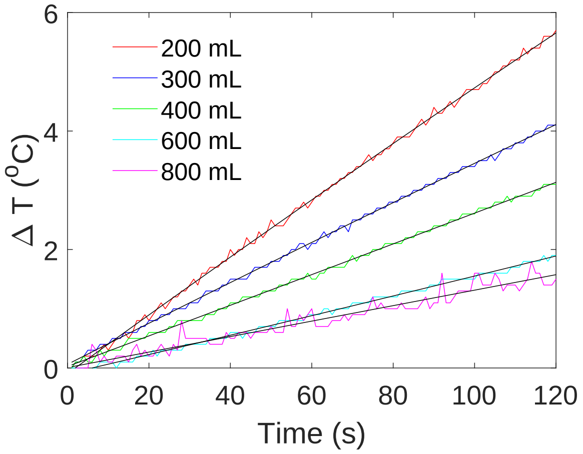

Figure S4: Temperature increase as a function of sonication time for different volumes of water. Black lines represent linear fits to the raw data.

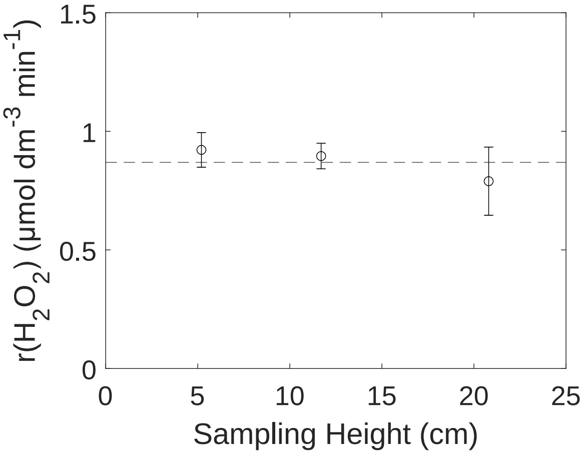

Figure S5: Rate of H_2O_2 formation as a function of sampling height for a total reactor volume of 800 mL. The mean value for all sample heights is indicated by the dotted line.

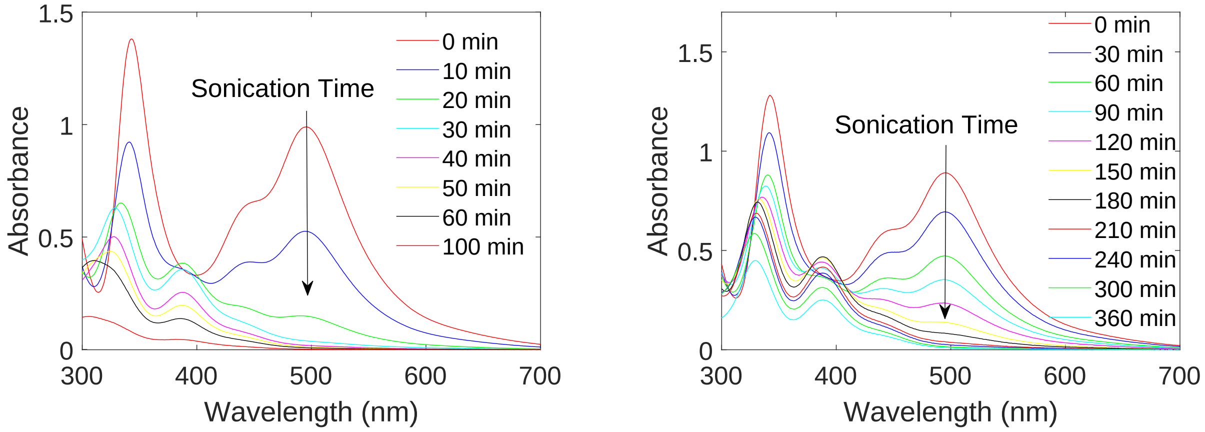

(a)

(b)

Figure S6: Absorbance spectra of $PtI_6{}^{2-}$ and $PtI_4{}^{2-}$ samples acquired at different sonication times with a solution volume of 200 mL (a) and 800 mL (b).
